# Effects of a Physical Activity Intervention on Physical Fitness of schoolchildren: The Enriched Sport Activity Program

**DOI:** 10.3390/ijerph17051723

**Published:** 2020-03-06

**Authors:** Ewan Thomas, Antonino Bianco, Garden Tabacchi, Carlos Marques da Silva, Nuno Loureiro, Michele Basile, Marcello Giaccone, David J. Sturm, Fatma Neşe Şahin, Özkan Güler, Manuel Gómez-López, Guillermo F. López Sánchez, Simona Pajaujiene, Ilona Judita Zuoziene, Ante Rada, Marianna Alesi, Antonio Palma

**Affiliations:** 1 Department of Psychological, Pedagogical, Educational Science and Human Movement, University of Palermo, 90144 Palermo, Italy; ewan.thomas@unipa.it (E.T.); antonino.bianco@unipa.it (A.B.); tabacchi.garden@libero.it (G.T.); marianna.alesi@unipa.it (M.A.); antonio.palma@unipa.it (A.P.); 2 CIEQV-Life Quality Research Centre, Escola Superior de Desporto de Rio Maior-IPSANTAREM, Av. Dr. Mário Soares, 20413 RIO Maior, Portugal; csilva@esdrm.ipsantarem.pt (C.M.d.S.); nunoloureiro@esdrm.ipsantarem.pt (N.L.); 3 University of Palermo Sport Center (CUS Palermo), Via Altofonte, 80, 90129 Palermo, Italy; miter702@libero.it (M.B.); marcello.giaccone@unipa.it (M.G.); 4 Department of School of Sport and Health Sciences, Professorship of Educational Science in Sport and Health, Technical University of Munich, Uptown Munich Campus D, Georg-Brauchle-Ring 60/62, 80992 Munich, Germany; david.sturm@tum.de; 5 Department of Sport and Health, Faculty of Sport Sciences, Ankara University, Golbaşı Yerleşkesi Spor Bilimleri Fakültesi, Golbaşı, 06830 Ankara, Turkey; oguler@ankara.edu.tr; 6 Department of Physical Activity and Sport, Faculty of Sports Sciences, University of Murcia, Calle Argentina, s/n., 30720 Murcia, Spain; mgomezlop@um.es (M.G.-L.); gfls@um.es (G.F.L.S.); 7 Department of Coaching Science, Lithuanian Sports University, Sporto 6, LT-44221 Kaunas, Lithuania; simona.pajaujiene@lsu.lt (S.P.); ilona.zuoziene@lsu.lt (I.J.Z.); 8 Faculty of Kinesiology, University of Split, Teslina 6, 21000 Split, Croatia; arada@kifst.hr

**Keywords:** fitness tests, schoolchildren, exercise, warm-up, sporting activities, cognitive tasks, inhibition, working memory, shifting

## Abstract

*Background:* Physical fitness in youth is a predictor of health in adulthood. The main objective of the present study was to understand if an enriched sport activity program could increase physical fitness in a population of schoolchildren. *Methods:* In a sample of 672 children aged 10.0 ± 1.90 years, different motor skills were tested by the 1 kg and 3 kg ball throw (BT), the standing broad jump (SBJ), the 30 m sprint (30mS), the leger shuttle run (LSR), the illinois agility test (IGT), and the quadruped test (QT). Within the controlled-trial, the intervention group (ESA) underwent an additional warm-up protocol, which included cognitive enhancing elements, for 14 weeks while the control group continued with ordinary exercise activity. *Results:* A significant increase was present regarding the 1 kg and 3 kg BT, the SBJ, the 30mS, and the IGT, while no significant difference was shown regarding the QT and the LSR in the ESA group between pre and post intervention. In the control group, no differences were present for any test except for the QT and the LSR post-test. *Conclusion:* A 14-week structured physical intervention had moderate effects regarding throwing, jumping, sprinting, and agility in a sample of schoolchildren.

## 1. Introduction

Physical inactivity is widely recognized as a risk factor for illnesses in adulthood [1,2,3]. Health-promoting interventions, including enhanced physical activity and exercise programs showed promising results in different life stages [4]. An increase of physical activity is also associated to an increase in physical fitness [5], and such increased level of fitness has a positive effect on health [6]. Physical activity may act by increasing the functional capacity of the cardiovascular system, increasing nervous plasticity and musculoskeletal efficiency, which will have widespread positive effects on the health of human beings [7,8].

Different exercise activities represent an effective strategy to increase motor competence, targeting various components of the individual, such as aerobic, anaerobic, dual task, and motor coordination [9]. Another aspect regarding the effects of exercise, is the relation between physiological and psychological health [10], which underlines that cognitive functions play a role in maintaining and promoting health [11]. There is an increase in cognitive functioning and mental health and wellbeing [12] following exercise, especially in children, in which such activities are linked to neuroplasticity [13]. Three main cognitive functions have been seen to increase following physical activities: inhibition, working memory and shifting [14]. An example of such phenomenon can be seen either regarding structural adaptations, such as increase of the brains gray matter and volume [15,16], an increase in the level of neurotrophins [17], and increased brain blood flow [18]. Also, functional adaptations, such as modifications in the neuronal network [19], which have also been seen to increase academic achievement [20] and improve memory and attention [18,21] can be found. Hence, poorer fitness levels correlate to poorer cognitive abilities [22]. However, such increase in cognitive functioning has been seen to be greater in those populations with a poor physical fitness level or those displaying mental or physical disabilities [23]. The majority of the studies linking the increase in neuroplasticity and cognitive function towards exercise tend to consider aerobic exercise as the primary form of exercise modality able to influence cognitive function [13,18,21]. Vice versa, limited evidence is available, explaining the possible mechanisms able to increase physical fitness through the implementation of cognitive-oriented tasks.

A thoroughly studied aspect, however, is coordination. Jensen et al. [24] reports the effects of a coordination-enhancing protocol on spatial cognitive tasks, concluding that such training may be useful to individuals who use spatial cognition in their working activities. Furthermore, Chang et al. [25] studied the effects of coordination training in pre-school children on event-related potentials. These variables provide a measure of the brain’s response to external stimuli. After an 8-week intervention, an increase in the pre-frontal cortex activity was remarkable, highlighted by increased P3 amplitudes waves and reduced reaction times on a flanker task. Both studies underline the possibility that specific types of coordination training influence aspects of cognitive function. Besides, Roebers et al. found that the influence of 9-month coordination training on motor skill development in children aged 5-6 years accentuated an increase in motor skills linked to cognitive performance [26]. A positive correlation of coordination towards strength has also been evaluated [27]. Coordination tasks incorporated into strength training promote a moderate increase in isometric strength. Moreover, the participants of the study of Rutherford and Jones lifted significantly more weight during the required task.

Within the intervention of the ESA project, namely the Enriched Sport Activity program, we aimed to integrate physical and cognitive tasks through an enriched sport activity program, which mainly included coordination exercises as a form of an additional warm-up, before regular sporting activities [28]. The aim of this study is to understand the effects of an enriched sports activity program intervention that includes cognitive-enhancing elements to physical fitness in a sample of children from seven European countries.

## 2. Materials and Methods

### 2.1. Participants

The ESA program is an evidence-based exercise program cofounded by the Erasmus + Program of the European Union (Key action: Sport-579661-EPP-1-2016-2-IT-SPO-SCP). In order to understand the effects of ESA, a sample of 672 children aged 10.0 (± 1.9) was selected from seven participating countries of the program (Italy, Germany, Turkey, Spain, Lithuania, Croatia, and Portugal) [28]. The sample was composed of 379 boys and 293 girls (Aged 10.2 ± 1.8 and 9.8 ± 2.0, respectively) who were allocated in an ESA or control group (*n* = 368 and *n* = 304, respectively). All children were healthy and free of any disability or musculoskeletal, cardiological, neurological or respiratory diseases or dysfunctions. Before the inclusion of the children in the ESA program, a parent or legal representative of each child signed an informed consent. The study was conducted in accordance with the Helsinki Declaration (Hong Kong revision, September 1989) and the European Union recommendations for Good Clinical Practice (document 111/3976/88, July 1990). The Lithuanian Sports University’s Research Ethics Committee in Social Sciences approved the study with No 98 579661-EPP-1-2016-2-IT-SPO-SCP (2018-02-05).

### 2.2. Study Design

The participants completed a baseline (t1) and post-test evaluation (t2), which consisted of a 1 kg and a 3 kg ball throw (BT), a standing broad jump (SBJ), a 30 m sprint test (30mS), an illinois agility test (IGT), a quadruped test (QT), and a leger shuttle run test (LSR). Each test assesses different components of physical fitness [29]. The schoolchildren performed the LSR and IGT test once and the other four tests three times, of which the best result was considered in the analysis. The children conducted the tests in a random order. After t1, the children were randomly assigned to the ESA or control group, respectively. The ESA group underwent the ESA program, performing exercises linked to executive cognitive functioning, in particular, inhibition, working memory, and shifting, rather than performance-enhancing tasks. These tasks were carried out for 14 weeks, divided in 27 different training units implemented during a warm-up of a duration of 15 to 25 min prior to each participant’s structured physical activity [28,30]. The warm-ups were structured into a baseline phase that could last up to 10 min, and a stimulation phase which could last up to 15 min. The innovation introduced by the program consists of standardization of the warm-up sessions, enriched by cognitive stimuli, through the introduction of propaedeutic exercises that are suitable for several sports activities. The control group instead, did not undergo the ESA intervention and continued with each own regular exercise or PE. After the interventions period, the participants were re-evaluated.

### 2.3. Intervention Procedure

Before the start of the project, each participating coach received training regarding testing procedures and training modalities. The training for each coach was individually carried out by each country adhering to the project. Training procedures and modalities were standardized and shared by each country. The training had a duration of 8 h, delivered within a single day, which comprised a theoretical and a practical part. The ESA program had a duration of 14 consecutive weeks in the context of schools and sport centers. The intervention involved children from 7 to 14 years of age who were physically active. The protocol consisted of an additional warm-up performed before the practiced PE of each participant. The participants of both the ESA and control group were children regularly practicing school physical education or different sporting activities such as basketball, soccer, handball, and volleyball. Therefore, the ESA group added the following procedure (the ESA program) to the practiced physical activity, while the control group did not. The control group continued practicing his/her activity without the additional warm-up phase. For each school or sporting center, there were children allocated in the ESA or control group. The trainer of both groups for each school or sport center was a trainer of the ESA program. The ESA program consisted of 27 units, each unit had a maximum duration of 25 min divided in a twofold phase: a baseline and a stimulation phase. The enriched activity was obtained by introducing stimuli belonging to both the cognitive and motor domain.

The cognitive elements for the stimulation phase could involve inhibitory control, working memory, or task-shifting skills. For inhibitory control, the coach gave verbal commands of an exercise to which corresponded the execution of another movement, before being associated. Concerning working memory, the coach orally explained an exercise series, and the child had to execute the exercise in reverse order. Regarding task shifting, the child performed the exercises following the instructor’s command, but when the instructor whistled, the athlete had to pass the ball to the child ahead. Concerning the validity of the proposed exercises, four internal experts (two sport scientists and two psychologists) qualitatively rated the exercises relatively to the congruence with the proposed executive function. All the discrepancies among their judgments were solved through discussion or the modification of the exercise, until an agreement was reached, before the start of the project.

The instructor had to whistle between three and five times for each circuit, allowing the athlete to vary at least once each exercise. For every domain, children had to complete a beginner level (B), then an intermediate level (I), and finally, an advanced level (A). Each level was composed of nine units. A series of coaches’ guidelines video-tutorials were recorded to maximize the protocol standardization across the European administrators.

### 2.4. Measures

*One kilogram and 3 kg ball throw:* The participant sat on the ground with the back against the wall and legs extended and apart. A valid straightforward throw of the medicine ball was performed by a quick extension of the upper limbs in full-range of motion parallel to the ground. The zero-end of the yardstick should be placed on the chest. Operators measured the distance between the zero-end of the yardstick on the chest and the landing point of the ball, detected by another operator, using a standard tape measure, to the closest 1 mm, from a line passing between the chair’s frontal legs to the point the ball landed touching the ground. The protocol required three attempts with the 1 kg and the 3 kg medicine ball each. The procedure was identical for the 1 kg and 3 kg ball.

*Standing Broad Jump:* The SBJ test was performed on a hard surface. The participants were in a standing position with heels on the starting line and feet parallel. The participants had to jump as far as possible in a horizontal direction. No indication had been given on the movement of the legs or the arms, so, the participants could perform a self-decided depth countermovement of the legs and perform a free-arm amplitude swing. The participants had to land with both feet together and block the jump without further advancement. The distance was measured using a standard tape measure, to the closest 1 mm, from the starting line to the heel of the closest foot to the starting line.

*Thirty meter Sprint test:* Participants were standing in a flying start position with both feet behind the starting line. The first operator (placed at the athlete’s back) gave the “ready” command (start command) and clapped his/her hands. After the start command, the athlete sprinted at maximum speed for 30 m. The second operator standing near the finish line started the stopwatch the moment the participant moved the rear support foot, and stopped it the moment the participant’s torso passed the finish line. The fastest time required to complete the task was considered for investigation. The measure was calculated through a regular stopwatch, measured in seconds to the nearest second decimal.

*Illinois agility test:* The standard procedure for the IGT was adopted [31]. The protocol consisted of a testing space of 10 × 5 m marked with cones, with four center cones spaced 3.3 m apart and four corner cones positioned 2.5 m from the center cones. The participant laid on the ground, hands by the shoulders, and all body parts behind the starting line. An operator (placed at the athlete’s back) gave the “ready” command (start command) and clapped his/her hands. After the start command, the participant got up quickly and ran through the course. At the finish line, the second operator recorded with a regular stopwatch, in seconds to the nearest second decimal, the time passed between the clap of hands and the moment in which the athlete’s chest passed the finish line.

*Quadruped test:* The participant was in all-fours position with both hands on the ground and the buttocks high in the air behind the starting line. An operator (placed at the athlete’s back) gave the “ready” command and clapped his/her hands. After the start command, the participant quickly proceeded forward, for a total distance of 10 m, alternating the diagonally opposite upper and lower limbs, e.g., right hand and left foot, and took steps until touching the finish line with one hand. The second operator waiting near the finish line started the stopwatch with a clap of hands and stopped the moment the participant touched the finish line with one hand. The measure was calculated through a regular stopwatch, in seconds to the nearest second decimal.

*Leger shuttle run test:* The standard procedure for the LSR was adopted [32]. At the “go” of the instructor, the participant had to run between two lines set 20 m apart at a pace dictated by a recorded tone at appropriate intervals. Velocity was 8.5 km·h^−1^ for the first minute, which increased by 0.5 km·h^−1^ every minute thereafter. The test was completed when the participant was not able to keep the rhythm, not arriving at the cones, two times in a row. Operators ensured that the participant correspond to the pace within the two beep signals. The number of stages was recorded, and the total number of completed stages was retrieved for investigation.

### 2.5. Statistical Analysis

Means and standard deviations were described for the total sample and for both the ESA and control groups. All data has been tested for normality using the Kolmogorov–Smirnov test. Since the data appears to be not normally distributed, non-parametric evaluation has been carried out. In order to identify differences between groups, the U-Mann Whitney test for unpaired data has been performed, whereas to identify differences within groups, the Wilcoxon Test for paired data has been performed. Cohen’s d, to estimate effect sizes, were also calculated. All tests were carried out with IBM SPSS Statistics (Version 25, IBM Corp., Armonk, NY, USA). Significance level was set to *p* < 0.05.

## 3. Results

Table 1 provides descriptive measures of the sample for boys, girls, and both groups. The table also reports means and standard deviations of t1 and t_2_ of both the ESA and control group, respectively.

Differences between conditions have been calculated for each test at t1. No difference was observed for any test except for the QT and LSR (*p* < 0.05, d 0.27 and *p* < 0,001, d −0.19, respectively). After the intervention, a significant difference in the performance measures of the 1 and 3 kg BT (*p* = 0.003, d −0.27 and 0.008, d −0.22, respectively), in the SBJ (*p* = 0.001, d −0.23), the 30mS (*p* = 0.014, d 0.11) and the IGT (*p* = 0.0112, d 0.12) in the ESA group was observed. No difference was present in the OT test and the LSR (*p* = 0.276, d 0.01 and *p* = 0.821, d −0.18, respectively). An opposite trend was seen in the control group in which no difference was observed in the 1 and 3 kg BT (*p* = 0.073, d −0.18 and *p* = 0.275, d −0.11, respectively), in the SBJ (*p* = 0.625, d −0.05), the 30mS (*p* = 0.692, d −0.01) and the IGT (*p* = 0.162, 0.06). However, a significant change was present in the QT and the LSR (*p* < 0.001, d 0.12 and *p* = 0.001, d −0.30, respectively). Table 2 presents statistical differences between t1 and t2 of both the ESA and control Group. No differences were present at t2 between conditions for any test.

## 4. Discussion

Our study aimed to understand the effects of an enriched sport activity program in the form of a structured warm-up with different levels of difficulty on physical fitness in a population of children. Our main results suggest that the fitness tests proposed increased their measure, whereas no increase was present in those tests pertinent to coordination and aerobic fitness. Conversely, the control group increased only in the coordination and aerobic fitness tests. However, little to no effect was present in both the ESA and control group between t1 and t2. The results obtained suggest that from a perspective of motor performance, the ESA activity does not produce highly significant effects. Therefore, the results are similar to those obtained from a regularly practicing physical activity group.

The ESA program at this stage is a first approach of an experimental exercise-based approach that aims to promote cognitive activity. Notwithstanding the fact that the observed effect does not provide a large magnitude, no negative effects within the intervention group were observed.

The level of physical activity has been well established to influence the level of physical fitness [33], and a physical activity dose-response has been inversely associated to health risk outcomes. In accordance, our results highlight that the majority of the tests proposed increased their measure, which can translate to an increase of the general physical fitness. The form of increased activity which was administered in this project was through structured warm-ups prior to each children’s ordinary activity. There have been various attempts to evaluate the influence of specific warm-up protocols on performance. A study by Yanci et al., [34] evaluated three different duration warm-up protocols (8, 15 and 25 min) in soccer players, highlighting that longer duration protocols were associated to an increased perceived exertion before the physical performance. A performance reduction was also observed during a 10 m sprint and no difference from the baseline evaluation was evinced in a modified agility test. This may provide an explanation regarding the results of the QT, which has also been proposed for a distance of 10 m. Comparably, neither of the test measures provided for our test show an increase after the intervention period. Another study has evaluated the influence of specific and general warm-ups on explosive muscular performance [35], resulting that only those pertinent to explosive strength provided additional value to the muscular outcomes. This aspect has also been highlighted by van den Tillaar et al., [36], who also stressed that during a warm-up, specificity should be preferred rather than duration. There may be a twofold explanation on why the LSR did not show an increase in the ESA group: Firstly, no specificity was provided regarding aerobic activity. Secondly, the duration of the warm-up activity was too long causing negative effects on the aerobic outcomes in the sampled population. Therefore, coaches should not provide excessively long warm-ups [36].

The implementation of the intervention was not controlled. This fosters the need for high-quality process-evaluation measures to assure the effectiveness of the ESA program intervention. However, our results may provide insight for future studies regarding our testing procedures.

The present study is not without limitations. During the intervention period, the school children all regularly practiced a sport activity. The different training schedules (volleyball, basketball, soccer, and handball) in both the ESA and control groups may have influenced the test results. Furthermore, we did no control for age-related influences, which may have affected the outcome measures. We want to point out that this study provides a tentative interpretation of intervention effects on physical fitness, since it remains unclear if the improvement of physical fitness evinced in the ESA group is due to the benefits of the ESA program, due to the regular exercise practiced, or for the developmental phase of the participant’s life. Finally, the validity of the enriched activities was controlled only from a qualitative point of view. Such limitations may represent important information for future projects that will aim to use exercise-based approaches to implement cognitive activities.

To our knowledge, this is the first study to evaluate the effects of an additional structured warm-up session to ordinary sport activities in children. For this reason, we believe it is important to consider these data as a starting point, notwithstanding the small effects provided. Further application of the test battery would establish the research methodology and enhance the normative comparability of the sample. The ESA program has provided a tool that can be included in physical education classes or sport facilities as a strategy to promote physical fitness through coordination exercises and cognitive-enhancing elements. Another aspect that needs to be considered is that during the entire period of the ESA program (14 consecutive weeks), no children were either injured due to the proposed activity nor dropped out due to excessive training intensity, also reporting a generally high level of enjoyment. The ESA intervention is an easy-to-administer, enjoyable strategy to further promote physical fitness, which can be incorporated in a variety of sport activities and exercises of children.

## 5. Conclusions

The present study shows that an enriched sport activity, in the form of structured warm-ups, in addition to each children’s practiced physical activity, was able to promote some aspects of physical fitness among schoolchildren. The results obtained by the children who underwent the ESA intervention are in line with those that were regularly practicing physical activity.

An additional warm-up protocol may represent an effective strategy to include in ordinary workouts in order to promote physical fitness in youth. In conclusion, our methodology is the first structured exercise-based approach to include cognitive-enhancing elements for inhibition, working memory and shifting during other physical activities.

## Figures and Tables

**Table 1:** Descriptive characteristics of the sample.

Variable	ESA	Control	Total	
*n*	368	304	672	
	Boys	Girls		
*n*	379	293	672	
Age	10.2 ± 1.8	9.8 ± 2.0	10.0 ± 1.9	
**Tests**	**ESA t** **1**	**Control t** **1**	**ESA t** **2**	**Control t** **2**
1 kg BT (m)	3.00 ± 0.8	3.27 ± 1.2	3.22 ± 0.9	3.48 ± 1.3
3 kg BT (m)	1.93 ± 0.5	2.17 ± 0.7	2.05 ± 0.5	2.26 ± 0.8
SBJ (m)	1.38 ± 0.2	1.40 ± 0.3	1.44 ± 0.2	1.42 ± 0.3
30mS (s)	6.11 ± 0.8	6.17 ± 1.0	6.02 ± 0.9	6.20 ± 1.2
IGT (s)	21.2 ± 3.9	20.5 ± 5.5	20.8 ± 4.1	20.2 ± 5.3
QT (s)	6.7 ± 4.1	7.9 ± 5.1	5.9 ± 2.2	5.8 ± 2.4
LSR (stage)	33.1 ± 14.9	29.9 ± 16.8	35.6 ± 16.3	35.5 ± 19.4

The intervention group (ESA); ball throw (BT); standing broad jump (SBJ); 30 m sprint test (30mS); illinois agility test (IGT); quadruped test (QT); leger shuttle run test (LSR); m = meters; s = seconds.

**Table 2:** Statistical differences between t1 and t2 of both the ESA and control group.

Tests	ESA	Control
1 kg BT	0.003 *	0.073
3 kg BT	0.008 *	0.275
SBJ	0.001 *	0.625
30mS	0.014 *	0.692
IGT	0.011 *	0.162
QT	0.276	<0.001 *
LSR	0.082	0.001 *

Wilcoxon test for paired data was performed. * significant *p* < 0.05.
